# Internal Iliac Vein Reflux: An Underrecognized Pathophysiology in Klippel-Trénaunay Syndrome With Pelvis Involvement

**DOI:** 10.7759/cureus.21472

**Published:** 2022-01-21

**Authors:** Huaijie Wang, Chong Xie, Weilong Lin, Jinbang Zhou, Weijia Yang, Zhengtuan Guo

**Affiliations:** 1 Department of Pediatric Surgery, Xi’an International Medical Center, Xi'an, CHN

**Keywords:** surgery, dysmenorrhea, bleeding, hematuria, internal iliac vein, klippel-trénaunay syndrome

## Abstract

Background and objective

Internal iliac vein reflux (IIVR) has been underrecognized in Klippel-Trénaunay syndrome (KTS) with pelvis involvement. In this study, our aim was to report clinical and radiological characteristics, modified phlebography technique, and diagnostic and treatment algorithm and outcomes of IIVR in KTS patients with pelvis involvement.

Materials and methods

KTS patients diagnosed between May 2014 and January 2021 were retrospectively analyzed. The medical records and imaging studies of all patients with KTS of the lower extremities were included and reviewed. KTS was defined as the triad of capillary malformation, venous malformation, and limb overgrowth. Pelvis involvement was evaluated with MRI. Phlebography was performed if IIVR was suspected. IIVR ablation and sclerotherapy were performed if IIVR was confirmed in KTS patients with external genitalia/perineum manifestation and bleeding. Patients were followed up via outpatient consultations. Episodes of gross bleeding were specifically investigated.

Results

A total of 211 patients with lower limb KTS diagnosed by our team were included in the study. Unilateral IIVR was diagnosed in 97 patients, and bilateral IIVR in two patients; 117 KTS patients were managed with radiological intervention and/or hybrid surgery by our team. Eleven patients underwent an IIVR ablation procedure due to recurrent bleeding from pelvic organs. Postprocedural complications included transient fever (n=2) and mild anaphylactic reaction (n=1). A small hyperpigmented scar at the incision and/or accessing site was noticed in patients receiving bleomycin during the procedure (n=6). Bleeding episodes and anemia resolved in all patients during the follow-up period. Correspondingly, the involved IIV and its tributaries were found to have disappeared on imaging during the follow-up.

Conclusion

IIVR is common in KTS patients, and it can cause bleeding from pelvic organs. Bleeding can be managed with IIVR ablation and sclerotherapy in KTS with pelvis involvement.

## Introduction

Klippel-Trénaunay syndrome (KTS) is typically characterized by a combination of slow-flow vascular malformations (capillary, lymphatic, and venous) affecting an overgrown lower limb [1]. However, it may occasionally affect the upper extremity or both legs. In patients with KTS, the involvement of the gastrointestinal (GI) and/or genitourinary (GU) tracts is common, which can be a source of significant morbidity and even mortality [2]. In a 2007 study, the incidence of GU manifestations in KTS was about 30% [2]. The incidence of pelvis involvement may be as high as over 30% in KTS. The clinical manifestations include hematuria, menometrorrhagia, dysmenorrhea, and anorectal bleeding, ranging from occult to massive, and life-threatening hemorrhages. The management of bleeding from a vascular anomaly is initially conservative. However, KTS patients with clinically significant hemorrhages usually require invasive forms of management [2]. The problem is confounded further when the rectum and other pelvic structures are involved, especially in young adults with genital tract involvement [2-3]. Endoscopic therapy has a limited role because of the commonly diffuse nature of the potential bleeding sites. Surgical resection with partial or complete excision of the involved organ is the mainstay of the management of bleeding as per the literature [2-7].

Our team used a modified phlebography technique to develop malformed veins involving the pelvis. We found that KTS patients with clinically GI and/or GU hemorrhage had the ipsilateral internal iliac vein reflux (IIVR). We assumed that IIVR may be an important cause of GI and/or GU tract bleeding. In light of this, we developed less invasive approaches to ablating the IIVR for the management of bleeding, including endovenous intervention and endovenous-based hybrid surgery. Encouraging outcomes were achieved with only minor complications in patients with bleeding. In this study, we report the clinical and radiological characteristics, modified phlebography technique, diagnostic and treatment algorithms, and outcomes of IIVR in KTS patients with pelvis involvement.

## Materials and methods

Study population

The records of all patients with KTS treated by our team between May 2014 and January 2021 were reviewed. The MRI data of all patients with KTS of the lower extremities were evaluated to detect pelvis involvement. KTS was diagnosed on the basis of clinical and imaging studies by our multidisciplinary team, including pediatric interventional radiologists, sonography experts, and pediatric surgeons. The diagnostic criteria were based on clinical, sonographic, and MRI studies. Institutional ethics board approval was obtained prior to the data collection. Informed consent was obtained from all participants. Episodes of bleeding and anemia were recorded for all patients with pelvis involvement based on documentation and anamnesis. Endoscopic examination, endovenous treatment, and hybrid surgery data were recorded for all patients with GI and/or GU tract bleeding.

Radiological evaluation

**Figure 1:**
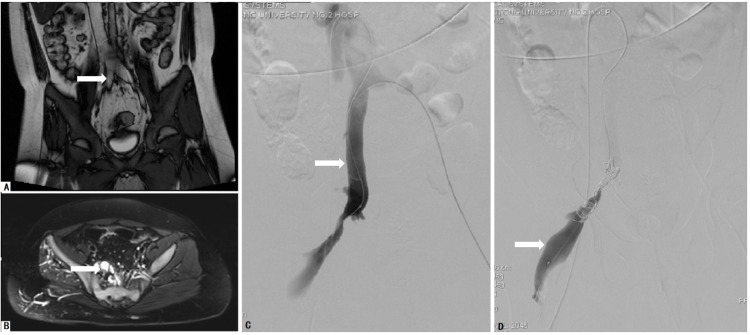
Images of a 12-year-old boy with KTS with gross anorectal bleeding who underwent trans-femoral vein radiologically interventional IIV embolization and sclerotherapy (FRIIVES) A: MRI revealed an aberrant and dilated IIV (arrow). B: The malformed IIV (arrow) showed a higher signal than the contralateral normal vein on the T2-weighted axial MRI scan, which implied IIV reflux. C and D: The malformed IIV (arrow) was managed with coils, ethanol, and foamed polidocanol for ablating bleeding IIV: internal iliac vein; KTS: Klippel-Trénaunay syndrome; MRI: magnetic resonance imaging

The pelvis is routinely evaluated in patients with KTS by MRI without contrast. If serial images demonstrated venous and/or lymphatic malformation (VM/LM) permeating the pelvic organs, including the anorectal region and the urinary or genital tract, the involvement of the pelvis could be confirmed. Among these patients, if serial T2-weighted axial MRI scans of the pelvis revealed that the internal iliac vein (IIV) had a higher signal than the contralateral normal vein (Figure 1), the IIVR was considered a potential diagnosis by our multidisciplinary team. In patients evaluated by magnetic resonance venography (MRV), we considered a dilated IIV to be an indication of the presence of IIVR [8]. The modified phlebography was recommended for these patients if IIVR was indicated in MRI or MRV.

Modified phlebography

The phlebography was performed under general anesthesia in a hybrid operating room. The dorsal venous rete of the foot was accessed to perform phlebography by compression maneuvers for checking the patency of the deep vein system and overviewing the lower limb vein system, including the femoral vein, great saphenous vein (GSV), marginal vein (MV) system, and possible persistent sciatic vein. If the absence/occlusion of the deep vein was confirmed by phlebography, aggressive intervention would be contraindicated. If the femoral vein was patent, GSV or femoral vein was accessed to perform phlebography to check whether the IIVR was present. The IIVR was confirmed if the reflux of the external-common iliac vein into IIV was observed.

Management strategy and follow-up

In this study, KTS patients with sigmoid colon involvement were excluded. In patients with KTS, the MV system was managed with transvenous ethanol embolization (TVEE) or combination with radiofrequency ablation (RFA) if conservative measures failed [9]. Failure was defined as progressive varicosity of the MV system or the presence of pain, or resistant hemorrhage causing anemia (hemoglobin <90 g/L). If IIVR was confirmed in KTS patients with external genitalia/perineum manifestation (phlebectasia, venous malformation, capillary malformation, etc.) or GI and/or GU tract bleeding, IIVR ablation and sclerotherapy were recommended.

**Figure 2:**
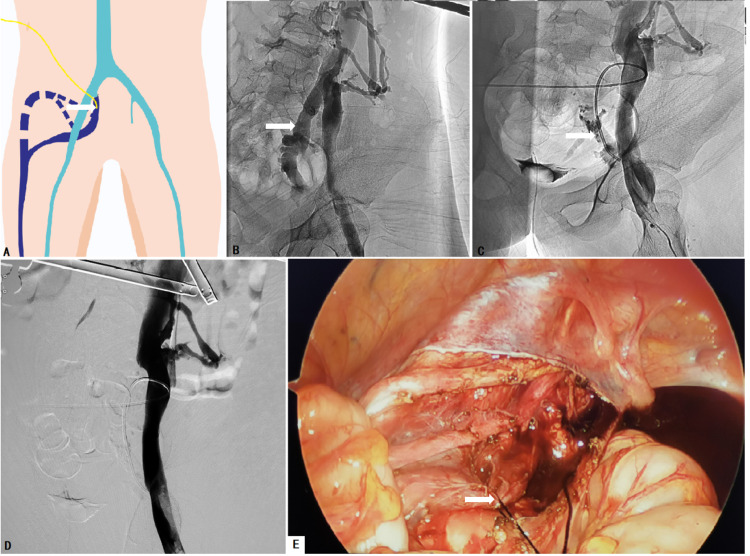
Laparoscopic internal iliac vein (IIV) ligation and trans-abdominal sclerotherapy (LILAS) A: The drawing of LILAS. The arrow shows that the catheter was placed in the IIV via abdominal wall access. B: Prior to operation, phlebography revealed IIV dilation and reflux (arrow). C: After laparoscopic IIV ligation, a catheter was introduced into the IIV (arrow) via abdominal wall access for ablating IIV and its tributaries. D: Phlebography confirmed that IIV reflux was successfully ablated. E: The malformed IIV (arrow) was ligated laparoscopically

**Figure 3:**
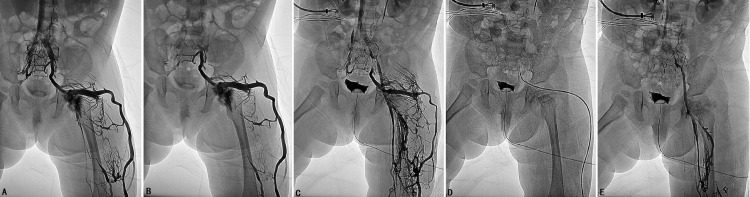
Images of a 15-month-old boy with KTS with gross anorectal bleeding who underwent laparoscopic IIV ligation and trans-marginal vein (MV) sclerotherapy (LIMS) A: Phlebography revealed that the MV directly connected to the IIV. B and C: After IIV ligation, phlebography showed that the IIV was occluded and the femoral vein was patent. D: A catheter was introduced into the IIV via MV access for ablating IIV and its tributaries. E: Phlebography confirmed that IIV and MV were successfully ablated IIV: internal iliac vein; KTS: Klippel-Trénaunay syndrome

**Figure 4:**
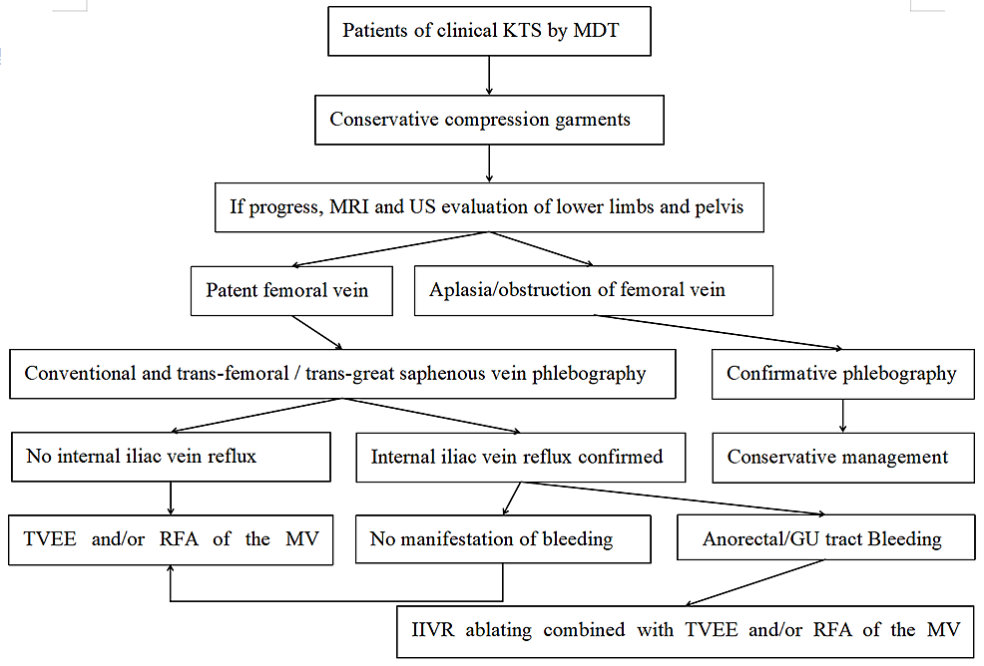
Flow chart of KTS management at our center KTS: Klippel-Trénaunay syndrome; MDT: multidisciplinary team; TVEE: transvenous ethanol embolization; RFA: radiofrequency ablation; MV: marginal vein; GU: genitourinary; IIVR: internal iliac vein reflux

Subcutaneous enoxaparin was administered for at least three days prior to the procedure (100 U/kg twice a day, or 150 U/kg daily). Four minimal invasive approaches were used to ablate the IIVR: trans-femoral vein radiologically interventional IIV embolization and sclerotherapy (FRIIVES) (Figure 1), laparoscopic IIV ligation and trans-abdominal sclerotherapy (LILAS) (Figure 2), laparoscopic IIV ligation and trans-femoral vein sclerotherapy (LIFS), and laparoscopic IIV ligation and trans-MV sclerotherapy (LIMS) (Figure 3). Sclerotherapy was performed with trans-catheter endovenous injection of ethanol and bleomycin and/or foamed polidocanol. Indications for different management modalities are described in Figure 4.

The procedure was performed under general anesthesia with endotracheal intubation following phlebography. These treatments were aimed at ablating refluent IIV and its tributaries, including malformed gluteal veins; VM involved the pelvis and potential bleeding sites, so as to ameliorate or eliminate symptoms, such as bleeding and anemia.

The postoperative complications were recorded. Patients were followed up via outpatient consultations: four weeks, three months, and six months after the procedure, respectively. Episodes of gross bleeding were specifically investigated.

## Results

A total of 211 patients with lower limb KTS diagnosed by our team with available pelvic MRI were included in the study. After analyzing the MRIs of these patients, unilateral IIVRs were considered in 97 patients, and bilateral IIVRs in two patients; 117 KTS patients were managed with radiological intervention and/or hybrid surgery by our team. Of those evaluated by pelvic MRI or MRV prior to the procedure, pelvis involvement and IIVR were respectively considered in 41 and 40 patients. Among these 41 patients, five were evaluated by MRV. The IIVR was confirmed by modified phlebography in all 41 patients. We failed to identify the IIVR on pelvic MRI due to concomitant extensive pelvis involvement of the LM component in a patient. However, the IIVR was observed during later phlebography. Modified phlebography showed the involved IIV was a valveless malformed vein in all these 41 patients.

Eleven patients underwent an IIVR ablation procedure because of recurrent bleeding from GI and/or GU tract. All 11 patients confirmed frequent episodes of gross visible bleeding. Five patients underwent colonoscopy examination. Colonoscopy revealed submucosal reticular phlebectasia of the rectosigmoidal colon. However, no active bleeding site was found. The age, affected side, bleeding manifestation, associated symptom, procedure, and used materials and sclerosant are summarized in Table 1.

**Table 1:** Patient information Sclerotherapy was performed with trans-catheter endovenous injection of ethanol and bleomycin and/or foamed polidocanol FRIIVES: trans-femoral vein radiologically interventional IIV embolization and sclerotherapy; LILAS: laparoscopic IIV ligation and trans-abdominal sclerotherapy; LIFS: laparoscopic IIV ligation and trans-femoral vein sclerotherapy; LIMS: laparoscopic IIV ligation and trans-MV sclerotherapy

Patient	Age	Affected side	Bleeding manifestation	Constipation	Procedure	Used materials and sclerosant
1	3 years	Left	Gross hematuria	Constipation	FRIIVES	Coils, ethanol, and bleomycin
2	12 years	Right	Gross anorectal bleeding	-	FRIIVES	Coils, ethanol, and foamed polidocanol
3	62 years	Right	Gross hematuria, dysmenorrhea, and anorectal bleeding	Constipation	FRIIVES	Coils, ethanol, bleomycin, and foamed polidocanol
4	19 years	Left	Menometrorrhagia	Obstipation	FRIIVES	Coils, ethanol, and foamed polidocanol
5	11 years	Left	Gross anorectal bleeding	-	LILAS	Ethanol and bleomycin
6	16 months	Right	Gross anorectal bleeding	-	LIMS	Ethanol and bleomycin
7	27 years	Right	Gross hematuria	Constipation	LIFS	Ethanol and foamed polidocanol
8	4 years	Left	Gross anorectal bleeding	Obstipation	LIFS	Ethanol and foamed polidocanol
9	15 months	Left	Gross anorectal bleeding	Constipation	LIMS	Ethanol and bleomycin
10	11 years	Left	Gross anorectal bleeding	Constipation	LILAS	Ethanol and foamed polidocanol
11	8 years	Right	Gross anorectal bleeding	Constipation	LIMS	Ethanol and bleomycin

**Figure 5:**
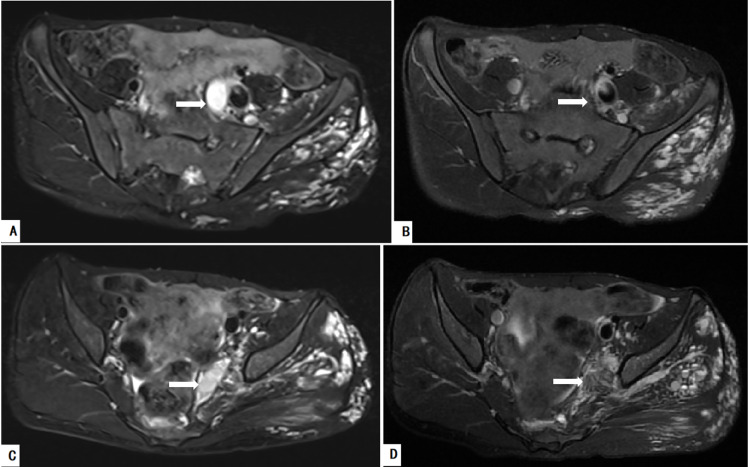
Detecting internal iliac vein on MRI scan preoperatively and during follow-up A and B: A dilated internal iliac vein (arrow) with a high fluid signal on T2-weighted axial MRI scan was successfully ablated (arrow). C and D: Malformed tributaries of the internal iliac vein (arrow) with a high fluid signal on T2-weighted axial MRI scan were also successfully ablated (arrow) MRI: magnetic resonance imaging

Postprocedural complications included transient fever (n=2) and mild anaphylactic reaction (n=1). A small hyperpigmented scar at the incision and/or accessing site was noticed in patients receiving bleomycin during the procedure (n=6). Bleeding episodes and anemia resolved in all patients during the follow-up period. Correspondingly, the involved IIV and its tributaries were found to have vanished on MRI scans during the follow-up (Figure 5).

## Discussion

This is the first report to study the IIVR in KTS patients with pelvis involvement, including MRI features, modified phlebography technique, ablation approaches, and treatment outcomes of bleeding. The incidence of pelvis involvement in our patients was 35.04% (41/117), which was higher than that in the literature [2]. Although the incidence is accurate for our patients, it should be noted that there is some selection bias with the most recalcitrant and/or difficult patients referred to our team for diagnosis and treatment. Furthermore, in KTS patients without genital and/or perianal cutaneous manifestations (phlebectasia, venous malformation, capillary malformation, etc.), the pelvis involvement may be underestimated. Therefore, we recommend that the pelvis involvement should be checked routinely with MRI without contrast axial T2-weighted sequence.

MRI was highly sensitive to detect the IIVR; the sensitivity was as high as 97.56% (40/41) in our patients. Identification of a refluent IIV in MRI depends on elaborately reviewing the T2-weighted axial scan of the pelvis, following along the course of IIV downward from the common iliac vein to gluteal veins. IIVR featured a typical fluid signal fulfilling the IIV and tributaries, including dilated gluteal veins. The signal is higher than that in the ipsilateral external iliac vein and contralateral normal veins. In our patients, only one IIVR was not distinguished by our team on MRI prior to phlebography. Postprocedurally, however, we retrospectively reviewed the MRI and identified the refluent IIV covered by extensive LM involving the pelvis. In KTS patients evaluated by MRV, IIVR featured a dilated IIV with a higher signal than normal. Nevertheless, MRV could not provide sufficient details to evaluate the extent of the pelvis involvement. Thus, we recommend MRI without contrast as the preferred imaging modality to evaluate the pelvis involvement in KTS patients.

Trans-femoral or trans-GSV phlebography was essential to confirm the presence of IIVR. In patients with KTS, there are usually three main superficial vein systems (great saphenous and small saphenous veins, and the MV system) and one deep vein system (femoral, popliteal, peroneal, and tibial veins) in the lower extremities. If the MV system is ascendingly connected to the IIV, IIVR would be difficult to identify on conventional phlebography. Thus, we modified the phlebography technique. Since blood from the “normal” superficial vein system (great saphenous and small saphenous veins) and deep vein system (femoral, popliteal, peroneal, and tibial veins) flow into the external iliac vein, if the IIV developed on phlebography via access to the femoral or great saphenous vein, IIVR can be confirmed. We recommend trans-femoral or trans-great saphenous vein phlebography for direct evidence to identify the IIVR.

We found that the IIV is a valveless malformed vein on phlebography in KTS patients with pelvis involvement. In some patients, the MV is ascendingly extended and directly connected to the IIV. Some MVs communicated to the IIV via gluteal veins. Similar to the MV in KTS patients, phlebography revealed that the involved IIV was avalvulia with reflux. In these patients, the IIV, gluteal, and MV form a complete valveless reflux system. In order to ablate the entire reflux channel, we divided it into two segments: the upper and the lower segment. The upper segment consisted of the IIV and gluteal veins. Four minimal invasive approaches mentioned above were used to ablate this segment: FRIIVES, LILAS, LIFS, and LIMS. The lower segment was the MV system below the level of buttocks. We preferred TVEE or a combination with RFA to manage this segment. RFA or transcatheter ethanol injection in gluteal veins should be avoided due to the risk of nerve injury. During transvenous ablating, placement of the tip of catheter/puncture needle for ethanol injection just superficial to or heating segment of RFA catheter just across the fibular head should be avoided due to the risk of common peroneal nerve injury [10].

The IIV and its tributaries represent interconnected networks of veins and venous plexuses draining the pelvic viscera, pelvic wall, external genitalia, perineum, buttocks, and medial thigh. Venous plexuses include the rectal, vesical, prostatic (in males), vaginal (in females), and uterine (in females) venous plexuses. IIVR can cause local venous hypertension of the ipsilateral draining area and phlebectasia of the involved viscera [11]. GI and/or GU tract bleeding is from hypertensive ruptured tributaries of IIV. Based on this concept, we believed that the IIVR plays an important role in the bleeding of KTS patients with pelvis involvement. Consequently, ablating the refluent IIV and its tributaries to decrease local venous tension and improve pelvic venous hemodynamics was emphasized in our procedures.

Nevertheless, bleeding was successfully controlled by ablating the IIVR in our patients. Corresponding to the hemorrhage disappearing during follow-up, the MRI scan revealed that the involved IIV and its tributaries had successfully vanished. Therefore, we can hypothesize that GI and/or GU tract bleeding in KTS is due to the local venous hypertension and rupture of tributaries.

In the literature, the management of bleeding for KTS patients included surgical resection with partial or complete excision of the involved organ [7], endoscopic sclerotherapy, endoscopic removal of the bleeding site [2], cauterization, and arteriographic embolization [12]. Partial resection of the rectosigmoidal colon was the mainstay of rectal bleeding [7]. Surgical excision always carried the risk of severe intraoperative bleeding and injury to adjacent structures. Repeated endoscopic sclerotherapies were needed since bleeding sites were usually diffuse. Refractory hematuria was treated with cauterization. These endoscopic approaches had a limited role because of the diffuse nature of the interconnected networks of veins and venous plexuses [5]. Furthermore, there was a risk of recurrent bleeding since local venous hypertension was not basically improved. Additionally, the MV connecting with or directly draining into the external iliac vein was not observed in our patients.

A primary limitation of this study was the small patient cohort. This small sample size was due to the low incidence of bleeding and underestimation of IIVR in KTS patients. Therefore, a prospective study to evaluate the role of IIVR in KTS may be necessary for further research.

In summary, we developed a new management concept to treat bleeding in KTS patients with pelvis involvement. The IIVR can be ablated with radiological intervention and hybrid surgery. For all treatments, the potential risks and benefits should be balanced carefully by a multidisciplinary team.

## Conclusions

IIVR is common among KTS patients. It is a form of congenital venous malformation, which can be detected on an MRI scan without contrast and further confirmed by trans-femoral vein or trans-GSV phlebography. In KTS patients with pelvis involvement, the IIV-gluteal-MV system is a valveless malformed reflux channel. IIVR can cause GI and/or GU tract bleeding, which can be managed by a hybrid multidisciplinary approach.
